# Top-Down Inference in the Auditory System: Potential Roles for Corticofugal Projections

**DOI:** 10.3389/fncir.2020.615259

**Published:** 2021-01-22

**Authors:** Alexander Asilador, Daniel A. Llano

**Affiliations:** ^1^Neuroscience Program, The University of Illinois at Urbana-Champaign, Champaign, IL, United States; ^2^Beckman Institute for Advanced Science and Technology, Urbana, IL, United States; ^3^Molecular and Integrative Physiology, The University of Illinois at Urbana-Champaign, Champaign, IL, United States

**Keywords:** auditory, cortex, thalamus, colliculus, top-down, speech perception, descending, medial geniculate body

## Abstract

It has become widely accepted that humans use contextual information to infer the meaning of ambiguous acoustic signals. In speech, for example, high-level semantic, syntactic, or lexical information shape our understanding of a phoneme buried in noise. Most current theories to explain this phenomenon rely on hierarchical predictive coding models involving a set of Bayesian priors emanating from high-level brain regions (e.g., prefrontal cortex) that are used to influence processing at lower-levels of the cortical sensory hierarchy (e.g., auditory cortex). As such, virtually all proposed models to explain top-down facilitation are focused on intracortical connections, and consequently, subcortical nuclei have scarcely been discussed in this context. However, subcortical auditory nuclei receive massive, heterogeneous, and cascading descending projections at every level of the sensory hierarchy, and activation of these systems has been shown to improve speech recognition. It is not yet clear whether or how top-down modulation to resolve ambiguous sounds calls upon these corticofugal projections. Here, we review the literature on top-down modulation in the auditory system, primarily focused on humans and cortical imaging/recording methods, and attempt to relate these findings to a growing animal literature, which has primarily been focused on corticofugal projections. We argue that corticofugal pathways contain the requisite circuitry to implement predictive coding mechanisms to facilitate perception of complex sounds and that top-down modulation at early (i.e., subcortical) stages of processing complement modulation at later (i.e., cortical) stages of processing. Finally, we suggest experimental approaches for future studies on this topic.

## Introduction

We effortlessly navigate a world filled with complex sounds. Despite challenging listening environments, such as having a conversation on a windy day, talking over a poor cell phone connection, or presenting a poster at a busy scientific meeting, the auditory system routinely extracts the meaning of signals corrupted by noise. One type of cue that may be used to perform this operation is the linguistic or acoustic context within which a sound exists. For example, it has long been known that high-level information about the nature of ambiguous speech sounds can dramatically enhance the ability to recognize these sounds (Miller et al., 1951; O’Neill, 1957; and reviewed in Davis and Johnsrude, 2007; Obleser, 2014). Also, acoustic perception and peripheral auditory responses in humans are strongly influenced by preceding non-speech acoustic stimuli (Lotto and Kluender, 1998; Skoe and Kraus, 2010), suggesting that contextual cueing may be a general mechanism used by the auditory system to deal with ambiguity. Contextual cueing is also of clinical importance as many individuals with language-related disorders, such as aphasia, autism, auditory processing disorder, and dyslexia, have difficulties using high-level contextual cues to disambiguate noisy or degraded sound stimuli (Tseng et al., 1993; Grindrod and Baum, 2002; Fink et al., 2006; Stewart and Ota, 2008; Chandrasekaran et al., 2009; Moore D. R., 2012).

The process of using prior knowledge to influence the processing of sensory information is referred to as “top-down modulation.” Originally described as “unconscious influence” by Helmholz in the 1800s (Von Helmholtz, 1867), top-down modulation is a ubiquitous process that is seen across all sensory systems (Kobayashi et al., 2004; Haegens et al., 2011; Andersson et al., 2018). It is believed that the major roles of top-down modulation are to select certain sensory features over others in a cluttered sensory environment to favor encoding information that is more meaningful for the organism. On the latter point, meaningful information is often defined by the statistical regularity with which those features are encountered in the environment, a key point exploited by most of the experimental paradigms involving repetitive stimulation of a particular region of cortex (e.g., Gao and Suga, 2000; Yan and Ehret, 2002).

The neural substrates for top-down modulation are not well understood. Sensory systems are hierarchically organized such that sensory information ascends through a series of brain regions before reaching the primary sensory cortex (e.g., the primary auditory cortex). Canonically, the primary sensory cortex sends projections to secondary sensory cortical areas, which then project to areas outside of the sensory pathway, typically including areas of the prefrontal cortex. Also, virtually all of these “ascending” connections are associated with a returning “descending” connection, which in some cases contain axons that greatly outnumber the corresponding ascending connection. In some cases, the descending connections “skip” levels and send projections to areas that do not have a direct corresponding ascending connection (e.g., the projection from the cortex to the tectum or to the corpus striatum). Virtually all current models that describe the use of top-down modulation to facilitate auditory processing have focused on intracortical projections [e.g., from the frontal cortex to auditory cortex or from secondary auditory cortical fields to the primary auditory cortex (Zekveld et al., 2006; Hannemann et al., 2007; Sohoglu et al., 2012; Chennu et al., 2013, 2016; Hofmann-Shen et al., 2020)]. What is often left out of the discussion, however, are the massive and heterogeneous projections emanating from the auditory cortex that target virtually every level of the subcortical auditory system (herein “corticofugal projections”) and, through cascading projections, impacting the most peripheral component: the cochlea (Xiao and Suga, 2002; León et al., 2012; Dragicevic et al., 2015; Jäger and Kössl, 2016).

**Figure 1:**
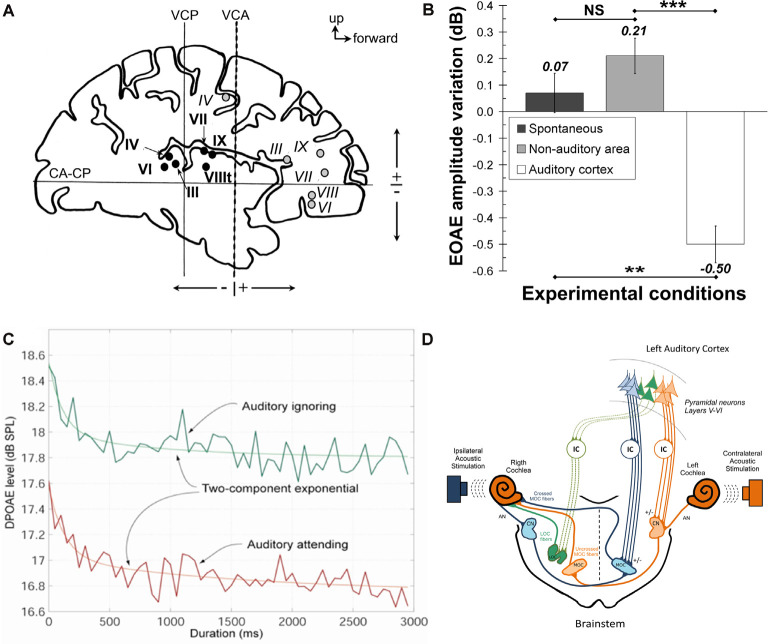
**(A,B)** Illustration of the experiment by Perrot et al. (2006) showing electrical stimulation sites in the human auditory cortex in panel **(A)**. Black circles = auditory cortex stimulation sites, gray circles = non-auditory cortex stimulation sites, Roman numerals correspond to the individual patients. CA-CP, plane passing through the anterior and posterior commissures; VCA, vertical plane passing through the anterior commissure; VCP, vertical plane passing through the posterior commissure. Panel **(B)** shows the change in the variation in the amplitude of evoked otoacoustic emissions (EOAEs) under spontaneous conditions (dark bar), after stimulation in the non-auditory cortex (gray bar), and after stimulation in the auditory cortex (white bar). These data illustrate that human auditory cortical stimulation diminishes the variability of evoked otoacoustic emissions. **P < 0.01; ***P = 0.001; NS, not significant using paired *t*-tests. Standard error of the mean is shown using error bars. Data obtained with permission from Perrot et al. (2006). Panel **(C)** Illustrates the impact of attending to an auditory stimulus on distortion product otoacoustic emissions (DPOAEs). As shown, attending to an acoustic stimulus diminishes the DPOAE amplitude (red trace), compared to ignoring that stimulus (green trace). Data obtained with permission from Smith et al. (2012). **(D)** Illustration of a model proposed by Terreros and Délano (2015)to explain the influence of the cortex on the cochlea. They propose multiple potential pathways from the auditory cortex, involving the inferior colliculus and the superior olivary nuclei, to impact the outer hair cells *via* the medial olivocochlear bundle. Figure obtained with permission from Terreros and Délano (2015).

This focus on cortical mechanisms of top-down modulation has existed despite the data demonstrating that descending influences can alter primary auditory input through the cochlear efferent system. For example, attentional tasks and prior linguistic knowledge modulate efferent projections to the cochlea (Collet et al., 1994; Marian et al., 2018), electrical stimulation of the human auditory cortex modulates cochlear activity (Perrot et al., 2006), and activation of subcortical auditory pathways to the cochlea facilitate speech recognition in challenging listening situations (De Boer and Thornton, 2008; Smith et al., 2012; Srinivasan et al., 2012; Mishra and Lutman, 2014; Shastri et al., 2014). As shown in Figures 1A,B, electrical stimulation of the human auditory cortex (but not non-auditory cortex) diminishes the mean amplitude and the variation in the amplitude of evoked otoacoustic emissions. Also, auditory attention leads to a decline in the amplitude of otoacoustic emissions, which are generated by the cochlea (Figure 1C). The projections from the auditory cortex that lead to modulation of the cochlea have been reviewed by Terreros and Délano (2015). They proposed a cascading model of multiple parallel pathways connecting the auditory cortex, inferior colliculus, cochlear nucleus, and superior olivary nuclei (including a direct projection from the auditory cortex to neurons making up the medial olivocochlear pathway; Mulders and Robertson, 2000) as potential neural substrates for these findings (Figure 1D; Terreros and Délano, 2015). Here, we attempt to link the bodies of literature on intracortical top-down modulation for processing of complex sounds (which has primarily been done in humans, with some notable exceptions; García-Rosales et al., 2020; Yin et al., 2020) and corticofugal modulation of subcortical auditory processing regions (which has primarily been done in animals), to develop a better understanding of the potential role of corticofugal projections in the disambiguation of corrupted acoustic signals.

## Evidence for Top-Down Modulation in The Auditory System: Human Studies

When engaged with acoustic stimuli, the goal at the behavioral level is the coherent perception of an object in its environment. In the auditory system, one of the earliest models used to describe perception was auditory scene analysis. The term was coined by Albert Bregman, a psychologist at McGill University (Bregman, 1994). He explored the idea that elements of a sound stimulus are grouped by the similarity of the components of a sound. These bottom-up features include the pitch, harmonicity, rhythmicity, similarity of sound, and timing of the sounds. Research in perceptual computing has shown some success in forming the foundation of scene analysis, where the computational model is capable of object detection, component extraction, and separation of sources in real-world situations (Smaragdis, 2001). However, when the level of ambiguity increases, object separation becomes much more difficult. Researchers have investigated the effect of attention to resolve ambiguities, such as the separation of objects from distractors and noisy environments. For example, van Noorden (1971) examined stream segregation by presenting pure tones, tone A and tone B, to listeners. The stimulus was presented as a sequence of alternating A and B tones, but every second B tone was omitted. The two tones differed by a pitch for each experiment, and this difference was distinguished as either a denoted “small,” “intermediate,” or “large” difference. For small differences, the tones were perceived as a single rhythm and result in the perceptual fusion of the two tones. For large differences, the resulting perceived sound led to a separation of the two sounds, where the A tone was presented twice as fast as the B tone. For intermediate differences, the listeners either perceived either a fusion or fission of the two sounds based on the subject, however, the subjects can influence what type they hear based on the instructions given to the subjects. Thus, attentional bias can determine the nature of a percept when ambiguous signals are presented.

The effects of top-down modulation on bottom-up processing are particularly notable during speech perception. Any given speech unit is not represented solely by the instantaneous components of sound (frequency content and intensity) but is a time-varying cognitive construct whereby a combination of phonemes or acoustical patterns are used to represent a unit of speech. The same speech sounds vary from speaker to speaker and speech sounds may change based on their preceding or following sounds (coarticulation; Moore B. C., 2012). Yet, listeners can understand phrases and dialogue from different speakers without difficulty. As outlined by Davis and Johnsrude (2007), this form of perception is experience-driven and is demonstrated from an analysis by Fodor and Bever (1965) on the inclusion of clicks in a speech, as seen in speakers of Sub-Saharan languages. Such psychoacoustic tests have revealed that the clicks are not perceptually heard in individuals who have not acquired this language. The argument here is that speech understanding is a perceptual process such that humans cognitively reorganize the acoustic input stream based on our experience with acoustic stimuli.

Several core perceptual processes are needed to effectuate speech perception in the face of widely varying sensory stimuli. One is categorical perception—the tendency to perceive acoustic stimuli as belonging to distinct categories despite having their stimulus properties vary on a continuum (e.g., perceiving a phoneme as either voiced vs. unvoiced despite having a gradual change in voice onset time). Another perceptual process that is key to understanding corrupted speech is a perceptual fill-in. In speech, this is typically referred to as the “phonemic restoration effect” (Warren, 1970) and describes the process of perceptually filling in noise-filled gaps in speech with the missing phoneme, analogous to filling in the contour of a partially obscured or partially-constructed visual object (e.g., Kanizsa objects). A third core perceptual process needed for speech processing is segmentation. That is, knowing the start and the stop of a meaningful acoustic signal. Generally, speech does not provide clear temporal demarcations between meaningful utterances, and these have to be inferred by the listener. Finally, stream segregation—the ability to perceptually separate different auditory objects whose waveforms are intermingled—is key to deciphering speech buried in noise. Although these core perceptual processes for speech understanding can potentially be explained solely *via* bottom-up processes (see Norris et al., 2000 for arguments in favor of a purely bottom-up approach to speech processing), as will be reviewed below, they are all strongly influenced by top-down factors.

Early evidence that lexical or semantic context could be used to facilitate the categorical perception of speech in noise was provided by Miller et al. (1951). They reported that the intelligibility of a word is enhanced when the appropriate context is provided. For example, the word “trees” buried in noise is more intelligible if it is preceded by the phrase “Apples grow on ____”. Later work established that this effect is present at the lexical level (Ganong effect) such that preceding phonemes could increase the intelligibility of subsequent phonemes in words compared to nonwords (e.g., “task” vs. “dask”; Ganong, 1980).

Non-auditory cues can also be used to facilitate categorical perception. For example, observing the mouth movements of a speaker or seeing a written representation of a word before the obscured sound both facilitated perceptual performance (Sohoglu et al., 2012, 2014; Getz and Toscano, 2019; Pinto et al., 2019). For example, providing a written example of a semantically-associated word (e.g., “MASHED”) before an acoustic representation of a word with ambiguous voice onset time (e.g., “potatoes”), facilitated the categorical perception of the initial consonant more than unrelated visual primes (Getz and Toscano, 2019). This use of cross-modal semantic priming modulated the earliest electroencephalography (EEG) peak examined by the investigators, the N1 peak, thought to be related to primary auditory cortex activation (Hillyard et al., 1973; Näätänen and Picton, 1987). Also, to compare the contributions of frontal vs. temporal cortex in a similar task, Sohoglu et al. found that use of written word prior information to disambiguate a vocoded speech sound was associated with inferior frontal gyrus activation using a combined EEG/magnetoencephalography (MEG) approach. In contrast, manipulations of the number of frequency bands available (thus increasing the bottom-up detail in the stimulus), activated auditory areas of the superior temporal gyrus (Sohoglu et al., 2012). These data are in line with a fronto-temporal hypothesis about descending control (Tzourio et al., 1997; Braga et al., 2013; Cope et al., 2017).

Concerning perceptual fill-in, the influence of context on phonemic restoration has been extensively examined, even from the earliest descriptions of the restoration phenomenon. For example, Marslen–Wilson demonstrated in 1975 that phonemic restoration was much more common when the target word was placed in the appropriate semantic and grammatical context and that the third syllable of a word was much more likely to be restored than the first syllable (Marslen–Wilson, 1975), suggesting that within-word context is an important cue. Expectation effects were also found by Samuel in 1981 who showed that words with a syllable replaced by noise were more likely to be reported as intact words if those words were incorporated into a sentence (Samuel, 1981). Samuel later (Samuel, 1997) showed that phonemic restoration introduced adaptation effects similar to those predicted by previous top-down models (e.g., the TRACE model; Mcclelland and Elman, 1986). More recently, it has been shown that the phonemic restoration effect remains intact despite voice discontinuities pre-and post- noise gap. That is, listeners were able to perceptually fill-in the gap despite the absence of spectral overlap between the pre-and post- gap voice, suggesting that other cues, such as linguistic context, are driving the filling-in phenomenon (Mcgettigan et al., 2013).

The third core perceptual process needed to disambiguate noisy speech is segmentation. Because most languages do not have clear acoustic demarcations separating meaningful utterances in speech, segmentation between words and sentences must be inferred (e.g., “mother’s cold” vs. “mother scold”), and thus represents a key component of top-down speech perception (Davis and Johnsrude, 2007). Indeed a common complaint among most learners of a new language is not knowing where words start and end. Multiple potential cues can assist in this segmentation, such as loudness (stresses on particular syllables), word knowledge, semantic context, etc. Mattys et al. found that when multiple conflicting cues were available, listeners relied on higher-level cues (e.g., sentence context) rather than lower-level cues (e.g., word stress). They proposed a hierarchical organization with lexical knowledge occupying the highest level and what they referred to as “metrical prosody” (syllable stresses) at the bottom (Mattys et al., 2005). Supporting the idea that word knowledge plays a role in lexical segmentation is the finding by Cunillera et al. (2010) that knowing a small number of “anchor” words in a novel language facilitated the ability to appropriately segment that language into meaningful units. Similar knowledge-based facilitation of segmentation of musical phrases has been observed, suggesting that top-down facilitation of segmentation may be a general property of the auditory system (Silva et al., 2019).

Another key requirement for inferring speech content under noisy conditions is the ability to separate competing sound streams. This process is multifaceted and involves both bottom-up cues (e.g., different pitch contours or spatial locations of different sources, as described above; Bregman, 1994) and top-down cues. Many investigators have established that bottom-up cues are sufficient to separate sound sources (often referred to as “sound streams”) when the physical characteristics of the sound sources are distinct (Scholes et al., 2015). However, when there is substantial overlap between them, as is often the case in a sound-cluttered real-word environment, top-down cues become critical. Several studies have been done using such cluttered stimuli and have presented a priming stimulus containing the target and have observed a marked improvement in identifying the target (Freyman et al., 2004; Jones and Freyman, 2012; Wang et al., 2019). For example, Wang et al. examined the ability to separate two simultaneously-presented spectrally- and temporally- overlapping talkers without spatial cues. The presence of the target sound played before the simultaneously-presented sounds greatly facilitated the recognition of the target. This recognition was also associated with increased phase-locking of the superior temporal gyrus and sulcus MEG signals to the speech envelope (Wang et al., 2019). These data suggest that in the absence of bottom-up cues to separate sound sources, knowledge-based cues can be used and that this knowledge modulates processing in areas of the auditory cortex.

A common class of paradigms to study the various perceptual processes involved in auditory top-down modulation in humans is the oddball or omission paradigm. Such paradigms typically involve repetition of a particular sound, followed by an “oddball” (e.g., AAAAB), or the absence of sound (e.g., AAAA_). This paradigm or variations of it (e.g., presenting a global deviant such as AAAAA in the setting of a long series of AAAAB stimuli) have been heavily employed in the neuroscience literature. Oddballs typically evoke a voltage change measured at the scalp known as the mismatch negativity (MMN). The presence of the mismatch negativity has been taken as evidence of a core component of predictive coding—prediction error—and has thus been promoted as evidence for top-down modulation in the auditory system. The mapping of MMN onto top-down processing mechanisms is still not clear. The presence of some forms of MMN (sensitivity to local, rather than global deviants) in sleep or under anesthesia (Loewy et al., 2000; Nourski et al., 2018), which would be inconsistent with an active inferential process, suggests that bottom-up effects (such as habituation to repeated stimuli) may play a role. More modern instantiations of the oddball paradigm comparing responses to local vs. global deviants have shown that global deviants may be more vulnerable to anesthesia (Nourski et al., 2018), suggesting that this form of predictive error may better reflect active top-down control mechanisms.

## Computational Principles of Top-Down Modulation

**Figure 2:**
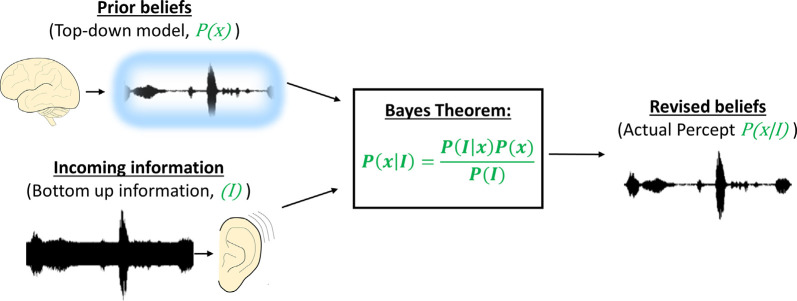
Illustration of the principles of Bayesian inference in neuronal coding. Top-down pre-conceived notions about a sound (illustrated as a sound trace surrounded by a blue haze) are combined with noisy information from the periphery (illustrated as a noisy sound trace entering the ear). Bayes theorem (in the box) combines the pre-conceived notions with the noisy sensory information to recover the original signal (here represented as the posterior probability or the actual percept.

Various models have been proposed to understand how contextual cues can influence sensory processing. Predictive coding is a general framework by which context, in the form of predictions about incoming data, can shape the properties of sensory-responsive neurons. Early instantiations of predictive coding algorithms were primarily focused on increasing the efficiency of the coding because of predictive coding’s ability to reduce redundancies in data streams (Srinivasan et al., 1982), similar to bandwidth compression required to transmit large images. Notably, efficiency in terms of the number of neuronal connections does not appear to be a design principle of descending systems in the brain. These systems are massive and typically dwarf ascending projections, so it seems unlikely that they evolved to maximize the efficiency of coding in lower centers. It is more likely that these large, presumably energy-expensive systems, evolved to increase the accuracy of identifying causes of sensory inputs. To this end, approaches that have been shown to increase the accuracy of sensory estimation, such as Bayesian estimation, have been postulated to be of use. Such schemes involve the generation of a prediction about the outside world (a Bayesian prior) that, when combined with noisy or degraded sensory information, leads to an optimal estimate of the cause of the sensory signal (the posterior probability), see Figure 2. The Bayesian priors are based on previous experience with the world and thus are updated by experience. Several studies have shown that in the setting of sensory uncertainties, humans combine contextual information and sensory information in Bayes-optimal ways (Jacobs, 1999; Ernst and Banks, 2002; Battaglia et al., 2003). More general models that attempt to explain neural processing based on similar principles (e.g., the Free Energy Principle) have been proposed (Friston and Kiebel, 2009). In practice, most predictive coding models involve a prediction, which is compared to sensory input. When the two are unmatched, a “prediction error” occurs (increasing the Free Energy), which is used as a learning signal to modify the internal model. This scheme is consistent with the large body of work showing enhanced neural responses to unpredicted stimuli (e.g., the MMN, reviewed above). However, as described in “Neural Models of Top-Down Modulation,” section this model has challenges both at the neural implementation level and at the level of linking neural responses to behavior.

Precisely how to link internal models with incoming information has been an open question. In modeling studies, the integration of predictive cues with incoming information has been implemented using several approaches. One approach has adapted linear systems theory and estimation theory into a model of the visual system. Rao and Ballard (1997) condensed the complexity of the visual system into a series of calculations that are inspired by work in minimum mean squared error estimation (MMSE): the Kalman filter (Kalman, 1960). The goal of MMSE is to estimate the internal (unknown) state of a system based on observation of noisy sensors to predict the next state. The Kalman filter is a linear estimator that assumes the noise from the environment is Gaussian. Further, any noise imparted by the internal state itself is pairwise uncorrelated to the noise of the sensor. This filter was used in an early model of hierarchical predictive coding in the visual system that, when trained on natural images, recapitulated some of the receptive field properties of early visual cortical neurons (Rao and Ballard, 1997). The model itself applies an extended form of the Kalman filter, capable of learning and prediction, with the learning rule obtained by the expectation-maximization (EM) algorithm formulated to mimic Hebbian learning. It can be shown that under Gaussian conditions that the Kalman filter is equivalent to the Bayes filter (Chen, 2003). A similar model to the extended Kalman filter initially proposed by Rao and Ballard (1997) has implemented a generative dynamical system in place of the Kalman filter to caputre nonlinearities of neural activation, and a learning scheme that takes into account the extra-classical effects experimentally observed in the visual system (Rao and Ballard, 1999).

Under non-Gaussian conditions, a more general implementation approach that has been used is the particle filter, as proposed by Lee and Mumford (2003). The calculations between the Kalman filter and particle filter are not similar, as the particle filter generates the likelihood weighting of states from the input and previous weights, followed by resampling of the input. While the Kalman filter is expressed by a linear operation, particle filters are constructed similarly to Markov chains to estimate the state of a given observation. The difference is that the dimensionality of the model is reduced by only looking at a weighted probability of being at a state instead of the total probability. This requires sampling a portion of the complete observation and estimating the weighted probability of the object being at some state. Subsequent re-sampling is performed with the weighted probabilities fed back into the model to more confidently estimate the state. In Lee and Mumford’s influential 2003 article, the authors introduce a concept of a particle filter-based model that hypothesizes that cortical connections are responsible for the calculations but interact in a way where each neuron represents specific events in the external world (i.e., features of an object; Lee and Mumford, 2003). It is described as a generative model, calculating the likelihood of the state hierarchically. Single neuron activation indicates a specific event in the external environment. The external environment shows the co-activation of specific patterns, and the state of the hidden variable depends on the state at the previous time step. Synchronized activity in a population of neurons contributes to the image. Here, the activity of superficial pyramidal cells correspond to the bottom-up messages, and the deeper pyramidal cells reflect top-down messages. Current state is conditionally independent of other past states.

## Neural Models of Top-Down Modulation

Neural models employed in predictive coding algorithms have relied heavily on descending connections between cortical areas. For example, in an early large-scale iterative model of visual cortico-cortical interactions that implemented predictive coding, a hierarchical network was proposed, with the lowest level focusing on a small portion of the image (local image patches) at a short time scale, and each subsequent level in the hierarchy representing increasing feature complexity such as larger spatial and time scales (Rao and Ballard, 1999). In this model, it was argued that each level in the hierarchy first starts at the inputs from the visual thalamus to the primary visual cortex (V1). Similarly, the hierarchical structure proceeds from V1 into the secondary visual cortex (V2).

Here, each level receives an input and estimates the object from its input. This estimate is calculated by a predictive estimator, learned from images the estimator is trained to, by a Kalman filter or generative system. It is argued in this model that this estimation is calculated *via* cortico-cortical connections in V1. Next, the model predicts the object at the next time step and conveys a predicted feature back to lower structures *via* descending cortical pathways. The usefulness of this hierarchical network model was established by its capacity to predict numerous types of neural and behavioral responses in the visual system. These include features such as: (1) distinguishing a learned image from occluding objects (i.e., bottle partially occluding an image of a hand) and background noise added to the image; (2) predicting a sequence of images; (3) end stopping; and (4) other “extra-classical” receptive field effects (Rao and Ballard, 1999).

**Figure 3:**
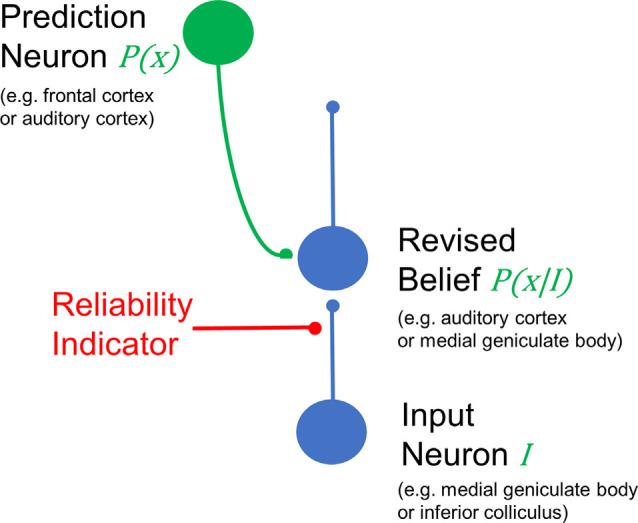
Generic example of the simplest circuit to involve top-down modulation to implement Bayesian predictive coding. A top-down projection (in green) carries the predictive signal [*P(x)*] from Figure 2. Such a signal could be derived, for example, from the frontal cortex or auditory cortex. This descending input is combined with weighted information from the periphery (represented as *I*) at an intermediate structure, such as the auditory cortex or medial geniculate body, using the examples provided above. Using this same scheme, *I* would be derived from the medial geniculate body or inferior colliculus. The weighting is determined by the reliability of the signal, conceived as a presynaptic input onto the input terminals. Neurophysiologically, this reliability signal could be represented by cholinergic or monoaminergic inputs that scale with arousal or attention. Note that this generic model is not limited to the structures listed on the figure, which are given as examples.

Establishing a neural implementation of predictive coding schemes has been challenging. At a minimum, one needs “prediction neurons” (or circuits) that provide a top-down signal and “prediction error neurons” (or circuits) that provide a bottom-up signal. In the context of the cerebral cortex, given the layer-specific directionality of cortical hierarchies (Rockland and Pandya, 1979; Felleman and Van Essen, 1991), prediction neurons would likely be found in the sources of descending connections: cells in layers 5 and 6. Since these cells project to layers 2 and 3 of areas lower in the sensory hierarchy, one would expect that supragranular layers would then contain prediction error neurons, as has been proposed previously (Bastos et al., 2012; Shipp, 2016). An important component of this basic circuit is the weighting of evidence from either bottom-up or top-down signals. For example, for highly reliable sensory signals, top-down predictions should carry less weight, while in situations of high sensory ambiguity (e.g., discerning a weak sound in noise), top-down signals should carry more weight. Most sensory systems do not have the luxury of repeatedly sampling the environment to determine the reliability of signals, but can estimate it based on saliency cues. It may be that neuromodulatory inputs (e.g., from cholinergic or monoaminergic fibers) can carry such a signal to dial up or down the reliance on top-down cues and thus adjust the “Kalman gain” of top-down modulation (Figure 3). Thus, sensory perception becomes a balance between reliance on top-down cues and bottom-up sensory saliency, as has recently been described human audition experiments (Huang and Elhilali, 2020). The over-reliance on top-down cues (possibly associated with disrupted neuromodulatory signals) may underlie pathophysiological states, such as the presence of delusions and hallucinations (Adams et al., 2014; Sterzer et al., 2018).

Physiological evidence for predictive coding at the single-neuron level has been observed in the visual cortex. Work in the late 1990s and early 2000s established that neurons in the early visual cortex of primates were sensitive to stimulus context and illusory signals (e.g., shape from shading or illusory contours in Kanizsa figures) and that these responses generally came after their response onsets (consistent with the time needed for feedback) and that the delayed responses were more characteristic of neurons from regions higher in the processing hierarchy (Lamme, 1995; Lee and Nguyen, 2001; Lee et al., 2002). Active silencing of descending connections from secondary visual areas can also eliminate surround suppressive effects, including end-stopping in V1 (Nassi et al., 2013), as proposed by Rao and Ballard (1999). More recent work has established similar patterns in the face-selective regions of the monkey temporal cortex (Schwiedrzik and Freiwald, 2017; Issa et al., 2018). In rodents, primary visual cortex neurons demonstrating responses to predictable stimuli, in advance of those stimuli, likely related to top-down signals from the cingulate cortex, have been identified (Fiser et al., 2016). These data all suggest that neurons in both the early- and late-visual cortex receive inputs from higher regions in the visual hierarchy that confer inferential properties upon those neurons.

However, applying these or other physiological data to a predictive coding model faces several challenges. First, as outlined above, cortical connectivity patterns in the primate brain imply that prediction neurons should be found infragranularly, and prediction error neurons should be found supragranularly (as has been proposed; Bastos et al., 2012; Shipp, 2016). Accepting the notion that we could recognize a “prediction neuron” when we see it (Kogo and Trengove, 2015), it is not clear from the physiological literature that there are differences in prediction error sensitivity in the upper vs. lower layers of the auditory cortex. For example, Atencio and Schreiner observed marked differences between granular and not-granular layers in terms of their representation of sound across multiple dimensions in the cat, but no indication that prediction-type neurons resided in infragranular layers or that prediction error was represented supragranularly (Atencio et al., 2009). Another study that observed suppression of motor-related prediction signals found that prediction error signals were found to be represented in the deep layers (Rummell et al., 2016), which is the opposite of that described by current canonical models of predictive coding (Bastos et al., 2012). Second, the general approach of subtracting away predictions implies that top-down projections should synapse on inhibitory neurons primarily—an idea for which there is little evidence—and that neural responses are smaller for predicted stimuli than for unpredicted stimuli. Regarding the former point, most work has revealed that descending intracortical projections form synapses on excitatory neurons and predominantly produce excitation (Johnson and Burkhalter, 1996; Shao and Burkhalter, 1996). Regarding the latter point, behavioral studies suggest that when ambiguous stimuli are congruent with expectations, behavioral performance is enhanced. Taken to its logical extent, the subtractive formulation of predictive coding implies that perfect predictions, which produce optimal behavior, *are associated with no neural responses*.

Most predictive coding schemes postulate that top-down predictions subtract from lower-level processors, leaving behind that which is not predicted—the prediction error. This scheme suggests that peripheral neurons are primarily responding to prediction errors—that which we do not predict. However, our behavior is just the opposite—we tend to ignore sensory data that do not fit into our predictions about the world. Thus, although predictive coding schemes that rely on the concept of prediction error can reproduce the responses of peripheral neurons, they do a poor job of explaining perception. We note that motor prediction may be a special case where subtraction is needed to remove the expected sensory consequences of actions (e.g., to suppress acoustic responses to vocalizations; Eliades and Wang, 2003), and here top-down motor-auditory circuits have been found to synapse on inhibitory interneurons (Nelson et al., 2013). More recent formulations have modified predictive coding algorithms to not include the subtraction operation for this reason (discussed in Spratling, 2017). Finally, predictive coding models have virtually ignored the massive sets of descending connections from the cortex that target subcortical regions, which have a very natural hierarchical organization. In the following sections, we explore the degree to which predictive coding models may be applied to the auditory corticofugal system.

## Early vs. Late Top-Down Modulation

**Figure 4:**
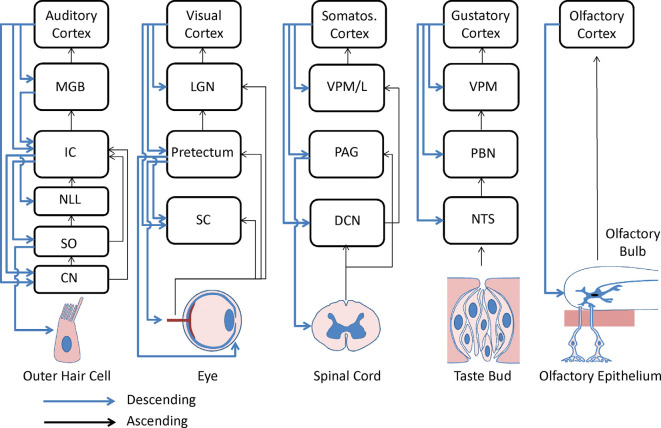
Diagram of known corticofugal and other subcortical descending projections across sensory systems. Black arrows, bottom-up projections; Blue arrows; top-down projections; CN, cochlear nuclei; DCN, dorsal column nuclei; IC, inferior colliculus; LGN, lateral genicular nucleus; MGB, medial geniculate body; NLL, nuclei of the lateral lemniscus; NTS, nucleus tractus solitarius; PAG, periaqueductal gray; PBN, parabrachial nuclei; SC, superior colliculus; SO, superior olive; VPL, ventral posterior lateral nucleus of the thalamus; VPM, ventral posterior medial nucleus of the thalamus. Taken with permission from Lesicko and Llano (2017).

As described above, most previous work on predictive coding in the auditory system has focused on the cerebral cortex. Corticocentric views of predictive coding have been driven by the fact that most of the relevant work on top-down modulation has been done in humans, where the techniques that are commonly used, EEG, MEG, and functional magnetic resonance imaging (fMRI), are most suited to measure activity in the cortex. Even though activity in subcortical structures may be seen in fMRI studies, they require appropriate hemodynamic response functions and often motion-correction procedures not needed for cortex, leading to the general absence of analysis of the subcortical activity in speech and language studies, as we have argued previously (Llano, 2013; Esmaeeli et al., 2019). However, there are massive projections to subcortical structures at all levels of the auditory system and these have been documented for at least 100 years (Held, 1893). For example, in the visual system (the only system to our knowledge where such an analysis has been done) descending projections from the visual cortex outnumber ascending projections to the thalamus by at least 3-fold (Erişir et al., 1997). Beyond descending control to the thalamus, there are projections from the auditory cortex to the inferior colliculus (Fitzpatrick and Imig, 1978; Winer et al., 1998; Bajo and Moore, 2005; Bajo et al., 2007; Bajo and King, 2013; Torii et al., 2013; Stebbings et al., 2014), from the thalamus to the inferior colliculus (Kuwabara and Zook, 2000; Senatorov and Hu, 2002; Winer et al., 2002; Patel et al., 2017), from the inferior colliculus to the superior olive and cochlear nucleus (Conlee and Kane, 1982; Caicedo and Herbert, 1993; Saldaña, 1993; Vetter et al., 1993; Malmierca et al., 1996; Schofield, 2001; Groff and Liberman, 2003) and from the superior olive to the inner and outer hair cells in the cochlea (Liberman and Brown, 1986; Guinan, 2006). Thus, manipulations at the level of the auditory cortex, *via* these cascading descending projections, can, and have been shown to, substantially influence processing at the level of the cochlea (León et al., 2012). Indeed, early work established attentional effects at the level of single units in the cochlear nucleus in cats (Hernandez-Peon et al., 1956). Analogous projections from the sensory cortex to the sensory periphery have been identified in other sensory systems as well (see Figure 4), suggesting that early filtering in sensory systems may be a general principle for top-down modulation.

Other investigators have proposed potential advantages to the application of top-down modulation at the early (subcortical) processing stage, rather than later (cortical) processing stages (He, 2003a). For example, seminal work by Broadbent suggested an early filtering mechanism based on the apparent loss of information that was ignored during a dichotic listening task (Broadbent, 1958). Modifications to this theory to account for some retention of information filtered at an early stage were also proposed (Treisman, 1964). Most recently, a “new early filter model” was proposed by Marsh and Campbell (2016) which postulated that long-range corticofugal-corticopetal (ascending) loops may be responsible for early filtering of signals at the level of the brainstem (Marsh and Campbell, 2016) and that a tradeoff may exist between early and late filtering depending on task requirements. For example, very challenging attentional tasks or tasks that require very rapid processing of information may be better suited for an early filtering process (Giard et al., 2000). Also, tasks that require filtering based on features that are lost as information ascends the sensory hierarchy (e.g., fine temporal structure) may also be optimally filtered before those representations being lost (Marsh and Campbell, 2016). Importantly, however, top-down modulation in speech processing occurs at multiple levels of abstraction and at multiple time scales, some requiring higher-level filtering. For example, top-down information may come in the form of lexical cues (operating over ms) or prosodic cues (operating over ms to seconds) as well as other dimensions, such as using low-level cues such as voice familiarity vs. high-level pragmatic cues (Obleser, 2014). Thus, late (cortical) and early (subcortical) modulation may play complementary roles in top-down modulation during active listening.

## Methodological Issues in Top-Down Modulation in the Subcortical Auditory System

Here we review methodological issues surrounding the study of descending projections from the auditory cortex to subcortical structures to effectuate top-down auditory control described above. It is worth noting that “descending projections” are not synonymous with top-down control. It is possible that lateral interactions within a brain structure (Srinivasan et al., 1982) can produce contextual modulation, as discussed in Rao and Ballard (1999) and Aitchison and Lengyel (2017). Here, we focus on corticofugal projections in keeping with the theme of this Special Issue on Cortical-Subcortical Loops in Sensory Processing.

Experimental paradigms for studying the corticofugal system have technical challenges that must be considered when analyzing the resulting data. Classical approaches include measuring response properties in a subcortical nucleus, then silencing the auditory cortex by cooling it or applying GABAergic agonists, and then re-measuring those properties. This paradigm is limited by: (1) incomplete recovery of cortical responses with certain GABAergic agents (Bäuerle et al., 2011); (2) lack of specificity of which layer (layer 5 or layer 6 corticofugal neurons) is silenced; (3) lack of specificity about which frequency ranges across the tonotopic axis of the auditory cortex are silenced; and (4) lack of knowledge if the effects of silencing are on the brain structure being studied (e.g., thalamus or inferior colliculus) or related to changes in the input to that structure from the cochlea, which is known to be impacted by cortical silencing (León et al., 2012). Regarding layer of origin, previous work has shown that both layers 5 and 6 project to the auditory thalamus and inferior colliculus (Games and Winer, 1988; Ojima, 1994; Künzle, 1995; Doucet et al., 2003; Bajo and Moore, 2005; Coomes et al., 2005; Llano and Sherman, 2008; Schofield, 2009; Slater et al., 2013, 2019), and that these projections have different physiological properties (Llano and Sherman, 2009; Slater et al., 2013) and likely different impacts on their target structures. Layer 5 cells have “driver”—type effects and layer 6 cells have “modulator”—type effects (for review see Lee and Sherman, 2010). Therefore bulk silencing is likely to homogenize the impacts of what could be quite different effects of these projections on their target structures. Likewise, work done using focal stimulation of the auditory cortex (reviewed in “Evidence That Auditory Corticofugal Systems Engage in Predictive Coding” section) suggests that corticofugal systems have markedly frequency-specific (in terms of the tonotopic axis) effects on their target structures, such that stimulation of neurons in certain frequency ranges can enhance, and others can suppress, subcortical responsiveness. Therefore, bulk silencing may produce a mixture of effects that are difficult to interpret. More modern approaches using viral-mediated delivery of optogenetic probes may solve some of these problems by permitting cell-type specific (Blackwell et al., 2020), layer-specific activation or silencing, and will permit activation or silencing to occur at the level of terminals, diminishing the likelihood of indirect effects stemming from changes in cochlear function.

Activating corticofugal projections with electrical stimulation has also been used in many studies, but also has potential methodological pitfalls. Specific to the auditory thalamus, electrical stimulation may antidromically activate thalamocortical neurons, which may then activate other structures, such as the thalamic reticular nucleus, whose neurons project back to the dorsal thalamus, leading to indirect effects. Importantly, the specific protocol of electrical stimulation may make a large difference in the impact on subcortical neurons. Small changes in the relative timing of cortical vs. acoustic stimulation, relative amplitudes, pulse rates, etc, can change responses from excitatory to inhibitory, even with optogenetic stimulation (Guo et al., 2017; Vila et al., 2019). Also, many studies have used stimulation paradigms that are really perceptual learning paradigms. That is, by repeatedly stimulating the corticofugal fibers and observing a change in tuning in a target structure, one is no longer only studying on-line modulation of sensory responses based on prior knowledge, but instead is studying the impact of tetanic stimulation of corticofugal fibers on synaptic plasticity in the target structure. Finally, much of the early work done on corticofugal modulation has been done on anesthetized animals. We know from work in human subjects that top-down projections appear to be particularly vulnerable to anesthesia or other factors that alter consciousness (Boly et al., 2011; Raz et al., 2014; reviewed in Sikkens et al., 2019), and thus may not be adequately studied in an anesthetized animal.

## Evidence That Auditory Corticofugal Systems Engage in Predictive Coding

The auditory cortex sends massive projections to the auditory thalamus (and related thalamic reticular nucleus), the inferior colliculus, and the cochlear nucleus. The projections to the thalamus and inferior colliculus emanate from layers 5 and 6, while those to the cochlear nucleus appear to only emanate from layer 5. It is not yet known whether there is a single layer 5 system that projects to all subcortical nuclei, though evidence exists for the presence of individual layer 5 cells that branch to the auditory thalamus and inferior colliculus (Asokan et al., 2018). Early work suggests that the layer 5 projections to the inferior colliculus and cochlear nucleus are independent (Doucet et al., 2003), though it should be noted that the double-backlabel technique used in this study is prone to false negatives if the two tracers are not placed into physiologically-matched zones in each structure. The layer 6 projections to the auditory thalamus and inferior colliculus are likely at least partially independent since they are found in different sublayers of layer 6 (Llano and Sherman, 2008; Slater et al., 2013; Stebbings et al., 2014).

The auditory corticothalamic system is massive, develops early, before hearing onset (Torii et al., 2013), elicits responses in the majority of MGB neurons (Ryugo and Weinberger, 1976; Villa et al., 1991; He et al., 2002) that are strong enough to induce immediate-early gene expression (Guo et al., 2007; Sun et al., 2007), produces both short (2 ms) and long (hundreds of milliseconds) latency responses (Serkov et al., 1976) and elicits both excitation (the dominant response in the lemniscal ventral subdivision) and inhibition (likely mediated *via* the thalamic reticular nucleus; Amato et al., 1969; He, 1997, 2003b; He et al., 2002; Xiong et al., 2004; Yu et al., 2004; Zhang et al., 2008). Activation of corticothalamic fibers can adjust tuning and sensitivity of auditory thalamic neurons (Guo et al., 2017) and appears to be critical for performance in perceptually-challenging tasks (Happel et al., 2014; Homma et al., 2017), as well as for directing plastic changes that occur in the thalamus (Zhang and Yan, 2008; Nelson et al., 2015). Importantly from the predictive coding perspective, corticothalamic projections appear to be organized topographically (Takayanagi and Ojima, 2006), such that cortical and thalamic areas that are matched for best frequency tend to produce corticothalamic excitation, while those that are unmatched tend to produce inhibition (He, 1997; He et al., 2002). Also, auditory thalamic neurons have been shown to be strongly sensitive to local stimulus predictability (Anderson et al., 2009; Antunes et al., 2010; Richardson et al., 2013; Cai et al., 2016), suggesting that they play a role in the coding of expectancy.

Several key experiments have been done to investigate the potential for corticothalamic fibers to contribute to predictive coding. One commonly-employed paradigm has been to apply repetitive stimulation of the auditory cortex to simulate a repeated acoustic motif and then to measure tuning properties to various parameters (sound frequency, combination-sensitivity, et cetera) before and after cortical stimulation. A consistent finding in the thalamus (and indeed in the inferior colliculus and cochlear nucleus, as described in the following paragraphs) is that stimulation of corticofugal fibers induces a shift of tuning of thalamic neurons towards the tuning of the particular region of the auditory cortex (so-called “egocentric selection”; Yan and Suga, 1996; Zhang et al., 1997; Zhang and Suga, 2000). From a Bayesian perspective, these data suggest that corticothalamic fibers contain “priors” such that the presence of highly prevalent stimuli (simulated by electrical cortical stimulation) makes it more likely that more peripheral responses in the thalamus, midbrain, or cochlear nucleus (i.e., posterior probabilities) are biased to respond more strongly to stimuli that are more likely to exist in the environment. The repeated stimulus presentation may be utilized to expand the cortical representation of Bayesian priors (Köver and Bao, 2010). As outlined in the “Methodological Issues in Top-Down Modulation in the Subcortical Auditory System” section, this paradigm falls short of establishing that corticothalamic fibers provide predictive coding signals because of the myriad problems with electrical cortical stimulation of the cortex, and because of the lack of establishment that acoustic stimuli use corticothalamic fibers to implement a predictive coding in the thalamus. Conversely, although it is well-established that training to alter the salience of an acoustic stimulus will shift neuronal tuning curves to be more responsive to that stimulus (Fritz et al., 2003), it remains to be established that the shift in tuning is caused by corticofugal projections.

An alternative approach has been to implement “surprise” paradigms, similar to MMN described in humans. The analogous finding at the single-unit level is known as stimulus-specific adaptation (SSA). In SSA, neurons diminish their responsiveness to repeated stimuli but retain their responsiveness to unexpected stimuli (Ulanovsky et al., 2003). Although it has been argued whether SSA is the neuronal-level instantiation of MMN (Farley et al., 2010; Carbajal and Malmierca, 2018), for our purposes, it is sufficient to state that SSA clearly reflects a key component of predictive coding: suppression of responses to predicted, presumably irrelevant stimuli. SSA has been established to exist in MGB neurons (Anderson et al., 2009; Antunes et al., 2010; Richardson et al., 2013; as well as neurons in the nonlemniscal inferior colliculus, below). Reversible silencing of corticothalamic fibers does not eliminate thalamic SSA, though it does alter other basic properties, suggesting that corticothalamic fibers play a strong role in modulating the thalamus, but may not confer SSA-sensitivity upon the thalamus (Antunes and Malmierca, 2011). We note that more aggressive nonreversible suppression diminishes thalamic SSA (Bäuerle et al., 2011), however, the significance of this finding is uncertain in the absence of reversibility of the cortical lesion.

We also note that the findings of SSA, and the paradigm employed by Suga and colleagues showing egocentric selection, are essentially orthogonal findings. That is, SSA represents the elimination of a predictable (presumably irrelevant) signal while the Suga paradigm represents the enhancement of a repeated, presumably behaviorally-important, signal. Evidence for both repetition suppression and repetition enhancement have been seen in the human subcortical auditory system (May and Tiitinen, 2010; Skoe and Kraus, 2010), though the latter is more in line with Bayesian notions of predictive coding. Thus, the data demonstrating egocentric shifts in thalamic receptive field properties suggest that corticothalamic projections may play an important role in providing a set of priors to thalamic neurons to bias their response properties, but may not be involved in repetition suppression manifesting as SSA.

The corticocollicular system emanates primarily from layer 5 of the auditory cortex with a smaller component from layer 6 (Games and Winer, 1988; Künzle, 1995; Doucet et al., 2003; Bajo and Moore, 2005; Coomes et al., 2005; Schofield, 2009; Slater et al., 2013, 2019), and primarily targets the nonlemniscal portions of the inferior colliculus, grouped here as the lateral cortex and dorsal cortex (Saldaña et al., 1996; Winer et al., 1998). In the lateral cortex, the auditory projections interdigitate with somatosensory projections in a manner that is determined by neurochemical modules present in the lateral cortex (Lesicko et al., 2016). Electrical stimulation of the auditory cortex produces collicular responses with latencies as short as 1–2 ms (Mitani et al., 1983; Sun et al., 1989) and produces both excitation and inhibition (Mitani et al., 1983; Sun et al., 1989; Bledsoe et al., 2003; Markovitz et al., 2015). The projections are tonotopic (Lim and Anderson, 2007; Markovitz et al., 2013; Barnstedt et al., 2015) and the inhibition is presumably at least disynaptic because the corticocollicular system is thought to be excitatory (Feliciano and Potashner, 1995), and the suppression occurs in the later phases of the response (Popelář et al., 2015). Corticocollicular fibers are responsible for protean functions at the level of the inferior colliculus, including facilitating adaptive changes in inferior colliculus neurons (Zhang et al., 2005; Wu and Yan, 2007; Bajo et al., 2010; Robinson et al., 2016; Asokan et al., 2018), sharpening of frequency tuning (Blackwell et al., 2020) and elicitation of escape responses (Xiong et al., 2015).

In terms of predictive coding, similar experiments to those done in the corticothalamic system have been done in the corticocollicular system but, in some cases, with a broader range of stimulus manipulations. For example, electrical stimulation of the auditory cortex causes egocentric shifts across multiple stimulus parameters, including frequency, duration, combination-sensitivity, sound location, and sound threshold (Yan and Suga, 1996, 1998; Jen et al., 1998; Ma and Suga, 2001; Yan and Ehret, 2001, 2002; Jen and Zhou, 2003; Yan et al., 2005; Zhou and Jen, 2005, 2007). These data suggest that the auditory cortex actively adjusts the tuning of collicular neurons to bias the response property across multiple computed stimulus dimensions and is not just inherited as part of the basic tonotopic layout of the two structures. Thus, a whole family of Bayesian priors (not unlike the family of hypotheses employed in particle filtering) can be used to modify the inferior colliculus. One challenge in understanding the corticocollicular findings is that most of the studies have involved recordings in the central nucleus of the inferior colliculus, which receives a small number of corticocollicular projections compared to the nonlemniscal regions. One potential resolution is that corticocollicular projections to the lateral cortex may have cascading inhibitory projections to the central nucleus after providing glutamatergic inputs to the lateral cortex, thus leading to primary inhibition in the central nucleus (Jen et al., 2001).

SSA has been observed in the dorsal and lateral cortices of the inferior colliculus (Malmierca et al., 2009; Duque et al., 2012), and it is thought that this is the earliest level that SSA occurs in the auditory system (Duque et al., 2018). Similar to the thalamus, reversible deactivation of the auditory cortex did not eliminate SSA in the inferior colliculus (Anderson and Malmierca, 2013). Thus, corticocollicular projections provide a strong predictive signal, possibly corralling inhibition from the lateral cortex en route to the central nucleus, to shift the tuning of collicular neurons towards those of previously heard stimuli. In contrast, suppression of repetitive irrelevant stimuli used in SSA appears to not involve these projections.

The auditory cortex also projects to the nuclei of the caudal auditory brainstem: cochlear nucleus, nucleus sagulum, and superior olivary nuclei (Feliciano and Potashner, 1995; Doucet et al., 2002; Meltzer and Ryugo, 2006), reviewed in Saldaña (2015). Compared to thalamic and collicular projections, comparatively little work has been done on these projections concerning predictive coding and all of it has been done in the cochlear nucleus. That said, all of the studies that have been done that measure tuning properties before and after focal cortical stimulation have revealed the same egocentric selection process described above for corticothalamic and corticocollicular neurons (Luo et al., 2008; Liu et al., 2010; Kong et al., 2014).

Notably, much of the early work on corticofugal modulation in animal models was done on echolocating bats (Yan and Suga, 1996, 1998; Zhang et al., 1997; Jen et al., 1998, 2001; Gao and Suga, 2000; Zhang and Suga, 2000; Ma and Suga, 2001). Although these mechanisms may be specific to echolocating bats due to their specialized behavioral requirements (Kössl et al., 2015), much of the key findings of the egocentric section have been seen in corticofugal projections non-echolocating species (Yan and Ehret, 2001, 2002; Yan et al., 2005; Luo et al., 2008; Liu et al., 2010; Kong et al., 2014). These data suggest that the basic principle of shifting tuning towards highly stimulated cortical representations is shared amongst both echolocating and non-echolocating species.

## Circuit-Level Mechanisms of Corticofugal Top-Down Control

Virtually all work to date on corticofugal modulation in the auditory system has been done at the level of phenomenology without circuit-level analysis. Interestingly, corticothalamic, corticocollicular, and corticobulbar projections all appear to have similar effects on their targets—they produce egocentric modifications of receptive fields after repetitive stimulation. This similarity suggests a common neural substrate may exist across these projections. The layer 5 corticofugal system is common to these projections, and thus may be a potential candidate. Layer 5 corticofugal neurons have similar properties across regions of the cortex. They are large pyramidal cells with long and tufted apical dendrites that burst intrinsically when depolarized (Connors et al., 1982; Kasper et al., 1994; Hefti and Smith, 2000; Llano and Sherman, 2009) and receive direct inputs from the thalamus (Constantinople and Bruno, 2013; Slater et al., 2019). In the corticothalamic system, these axons end in large terminals that synapse on proximal dendrites, producing “driver” type responses (Reichova and Sherman, 2004; Prasad et al., 2020). As described above, auditory corticothalamic terminals branch to the inferior colliculus (Asokan et al., 2018), but corticocollicular axons apparently do not branch to the cochlear nucleus (Doucet et al., 2003). In this respect, the layer 5 auditory corticothalamic system may diverge from other corticofugal systems where widespread subcortical branching is seen (Bourassa et al., 1995; Deschenes et al., 1996; Kita and Kita, 2012), reviewed in Usrey and Sherman (2019). Future work with sensitive tracers will clarify the extent to which a single auditory layer 5 “broadcast” neurons exist that send similar training signals to auditory thalamus, inferior colliculus and cochlear nucleus. Alternatively, given the homogenous nature of the changes seen across these three auditory nuclei, and the potential for auditory cortex stimulation to alter ascending information flow from the cochlea (León et al., 2012), these changes may be, in part, caused by alterations in shared ascending auditory information. We note that layer 6 projections to the thalamus are more numerous than layer 5 projections but tend to have smaller and more distal terminals (Lee and Sherman, 2010), and relay inhibition through the thalamic reticular nucleus (Lam and Sherman, 2010). Layer 6 corticocollicular projections also emanate from smaller neurons than layer 5 and have thinner neuronal projections and end in smaller terminals (Yudintsev et al., 2019). These data suggest that the layer 6 system may operate on a slower time scale, and is more likely to engage inhibitory interneurons, and thus may have a different set of functions than the layer 5 system that has yet to be identified.

**Figure 5:**
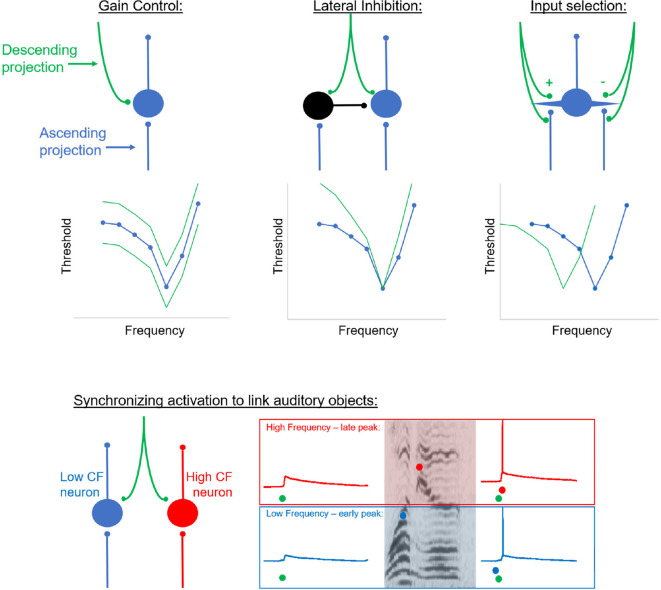
Putative circuit motifs that can implement top-down modulation of frequency receptive fields using descending projections. Green tuning curves represent the modified tuning curves after descending projections were activated. Left, the simplest “gain control” motif whereby top-down projections dial the responsiveness of target cells up or down, thus shifting the frequency tuning curve up or down. Middle, a lateral inhibition motif, whereby descending input inhibits inputs representing frequencies other than the characteristic frequency. In this case, the tuning curve would sharpen. Right, an input selection motif, where top-down inputs would either enhance (denoted with a “+”) or suppress (denoted with a “−“) certain classes of inputs either pre-or post-synaptically. In doing so, the top-down projections could eliminate inputs from what was the previous characteristic frequency and thus shift the tuning curve laterally. Bottom corresponds to descending inputs synchronously eliciting excitatory post-synaptic potentials (EPSPs) in two neurons of different characteristic frequencies. If a sound object has multiple frequency components peaking at different times (e.g., an early low-frequency peak and a later high-frequency peak, marked with blue and red circles on the spectrogram, respectively), then when those bottom-up inputs arrive at neurons with synchronized EPSPs, they are more likely to fire synchronous spikes, thus linking them as part of one auditory object. CF, characteristic frequency.

The synaptic mechanisms by which auditory corticofugal projections modulate response properties are unknown, but several limitations based on previous extracellular recording studies exist. For example, to effectuate a change in tuning to sound frequency, a significantly more sophisticated operation than “gain control” must take place. To induce a neuron to respond to a frequency of sound to which it was not previously responsive over a matter of minutes, there must have existed a population of latent (i.e., inactive) inputs that are responsive to those frequencies. Conversely, a population of synapses encoding previously-responsive sounds would need to be silenced. Although inhibitory/disinhibitory mechanisms may create these types of shifts and do appear to play a role in the corticocollicular system, a small fraction of the corticocollicular system (4%) synapses on inhibitory interneurons (Nakamoto et al., 2013). An alternative mechanism could be to strengthen or weaken synapses without the use of inhibition. Repetitive, tetanic stimulation of a focal area of the auditory cortex has been well-established to alter receptive field properties of that area of the cortex (Ohl and Scheich, 1997, 2005; Weinberger, 2004). Repetitive acoustic stimulation may also decrease the representation of that sound in the auditory cortex, depending on the behavioral salience of that sound (Condon and Weinberger, 1991). It is therefore possible that descending connections could strengthen synapses post-synaptically, though in the absence of an appropriately timed ascending signal would appear to be a non-Hebbian mechanism to induce a plastic change. Descending projections could also target presynaptic terminals to either activate them or diminish their strength, as suggested by early work in the visual system (Iwama et al., 1965), see Figure 5. However, at least in the auditory corticocollicular system, little evidence for presynaptic terminals exists in the corticocollicular or auditory corticothalamic system (Bartlett et al., 2000; Nakamoto et al., 2013). Beyond impacts at the level of individual cells, corticofugal projections may influence a population of cells to alter their likelihood of firing synchronously, as proposed previously (Gilbert and Li, 2013). Such a mechanism would be ideally suited to either integrate disparate pieces of information (as needed for contour integration, or phonemic restoration) or to segregate information (as needed during speech segmentation or stream segregation). For example, neural responses to a sound object with complex spectrotemporal properties with low and high-frequency peaks at different times may be linked into a singular perceptual object if descending projections synchronized subthreshold responses across an array of sensory neurons (Figure 5, bottom). Thus, unsynchronized responses from neurons with different characteristic frequencies at low levels of the hierarchy could be tagged as being derived from the same acoustic object by eliciting synchronized responses at higher levels of the hierarchy. Very little work of this type has been done, though it should be noted that inhibition of the corticothalamic system leads to greater synchrony of firing between thalamic neurons, suggesting that the corticothalamic system has the potential to enhance segregation between input streams (Villa et al., 1999). Similar findings were reported in the corticocollicular system by Nakamoto et al. (2010). Thus, multiple non-mutually exclusive synaptic motifs may help to explain the impact(s) of the corticofugal systems, and none have been systematically explored to date.

## Conclusions and Future Challenges

Top-down modulation is observed at the level of behavior and the level of the single neurons, and there is still much work left to be done to understand how these two levels of top-down modulation are linked. In our view, the weight of the evidence suggests that at least one role of descending projections is to modify receptive field properties to bias them towards frequently-occurring or highly salient stimuli. However, consistent with the anatomical and physiological heterogeneity of these systems, additional roles are possible. Complicating matters is the finding that these systems are often intermingled and individual projections may have more than one role. A challenge, then, in the field is how to design an experimental paradigm to identify the circuit motifs that produce top-down modulation and how they alter perceptual responses. The first step is to decide precisely what is being studied. The term “predictive coding” is broad enough to encompass many different types of processing. For example, the term is used to describe both the “explaining away” of expected and ignored stimuli as well as the enhancement of expected but obscured stimuli. As described above, the computations underlying these two processes are not the same and do not appear to be handled by the same circuits. The other challenging experimental question is deciding which level of top-down modulation is to be studied.

There are many “descending systems” in the auditory system and many types of tasks that require top-down modulation. Descending projections extend from the frontal cortex to the auditory cortex and on to the cochlea, with a stop at every auditory subcortical structure along the way. Presumably, certain descending projections should be important for high-level modulation (e.g., using discourse cues to understand an ambiguous word) vs. low-level modulation (e.g., having a loud sound diminish the sensitivity of the cochlea to subsequent sounds). An additional dimension is task difficulty. That is, difficult tasks may require multiple descending projections to be involved, thereby altering the stimulus representation as soon as it enters the brain, and others may be less challenging, allowing later filtering, thus permitting several stimulus representations to “coexist” in the brain before one being selected. Therefore, engaging in a systematic process to identify which pathway is engaged during which task would be a starting point for future investigators.

Also, to facilitate comparisons across studies, it will be important for future experiments to specify the type and level of predictive coding being studied. Also, although electrical stimulation paradigms have provided insights about predictive coding by demonstrating that repetitive activation of a particular region of cortex can change the filtering properties of more peripheral sensory neurons (reviewed above), these changes typically have been found after long-term (minutes) tetanic stimulation of the corticofugal projections, which is a crude approximation to altering the statistical likelihood of a particular sound appearing in the environment. A more convincing demonstration would be to show that the tuning of a particular neuron changes dynamically, and under particular behavioral contexts (similar to that seen in Caras and Sanes, 2017) when the likelihood that a particular stimulus occurs changes. Besides, one would also anticipate that prediction neurons would have their strongest impact when peripheral signals are weak (i.e., the Kalman gain would be highest under these circumstances). Consistent with this idea, previous work has shown that top-down modulation tends to be strongest in broadly tuned neurons [presumably neurons with ambiguous frequency representations (Vila et al., 2019) or when acoustic stimulus amplitude is weak (Jen et al., 1998)]. It may be the case that neurons that are broadly tuned to isolated sounds may be more sharply tuned in other contexts. Also, future work should emphasize paradigms that alter stimulus expectancy without altering stimulus probability in awake animals [as used in Cai et al. (2016)], thus removing the bottom-up cue of stimulus probability. Finally, one experimentally pragmatic benefit of studying corticofugal systems is the physical separation between the descending system and the physical structure under study, allowing the examination of responses in putative “prediction axons” (presumably corticofugal) compared to bottom-up signals. This type of approach has been used in two-photon imaging of the visual system, where presumed prediction neurons in the anterior cingulate were labeled and their response properties appeared to carry prediction signals (Fiser et al., 2016). The use of this set of approaches would get us closer to understanding the unusual connectivity patterns described by Lorente de Nó almost 100 years ago (Lorente De Nó, 1933):

“The conception of the reflex arc as a unidirectional chain of neurons has neither anatomic nor functional basis. Histologic studies…show the universality of the existence of plural parallel connections and of recurrent, reciprocal connections.”

Thus, a deliberate approach using techniques to interrogate populations of neurons in awake animals will permit the understanding of the logic of highly recurrent systems whose roles have remained obscure for nearly a century.

## Author Contributions

AA and DL both wrote the manuscript together. All authors contributed to the article and approved the submitted version.

## Conflict of Interest

The authors declare that the research was conducted in the absence of any commercial or financial relationships that could be construed as a potential conflict of interest.
